# The relationship between the mtDNA copy number in insulin-dependent tissues and markers of endothelial dysfunction and inflammation in obese patients

**DOI:** 10.1186/s12920-019-0486-7

**Published:** 2019-03-13

**Authors:** Larisa Litvinova, Pavel Zatolokin, Maria Vulf, Ilia Mazunin, Daria Skuratovskaia

**Affiliations:** 1 Immanuel Kant Baltic Federal University, Russian Federation Kaliningrad, Gaidara 6 st, Russia; 2 Department of Reconstructive and Endoscopic Surgery, Kaliningrad Regional Hospital, Kaliningrad, Russia

**Keywords:** Obesity, Type 2 diabetes, mtDNA, NO, Endothelial dysfunction

## Abstract

**Background:**

Mitochondria play a central role in the regulation of energy metabolism, and the biogenesis of mitochondria is enhanced by the action of nitric oxide (NO), which is the key signaling molecule in the regulation of vascular homeostasis. A disturbance in the regulation of energy metabolism can be a key reason for the formation of insulin resistance and type 2 diabetes mellitus. Moreover, mitochondrial dysfunction leads to oxidative stress, which increases the production of proinflammatory cytokines. In this regard, the aim of this study was to identify the relationship of the copy number of mtDNA in adipose tissue from different locations (subcutaneous adipose tissue (SAT), mesentery (Mes), greater omentum (GO)), liver biopsy samples and mononuclear blood cells (MNCs) with endothelial dysfunction markers (eNOS, ET-1, iCAM-1, vCAM-1, VEGF) and inflammatory mediators (TNF-α, IL-6, IL-8, CRP, leptin) in obese patients (body mass index (BMI) > 35 kg/m^2^) with and without type 2 diabetes.

**Methods:**

The study included 88 obese patients (BMI > 35 kg/m2) treated at the Kaliningrad Region Hospital. The control group consisted of 20 healthy donors. To measure mtDNA copy number we used droplet digital PCR. The concentrations of molecules (TNF-α, IL-6, IL-8, VEGF, eNOS, ET-1, iCAM-1, vCAM-1, VEGF) were measured in plasma using the following sandwich enzyme-linked immunosorbent assays (ELISAs). Quantitative determination of leptin was evaluated by flow-fluorimetry on a «Bio-Plex Protein Assay System». Statistical analysis and graphs were obtained in R Statistical Software (version 3.3.1).

**Results:**

The systemic character of chronic subclinical inflammation in obesity is established, and an increase in the level of endothelial dysfunction molecules was observed in the blood plasma. The levels of TNF-a, IL-6, and IL-8 were positively correlated with increases in BMI, serum glucose and cholesterol levels.

**Conclusions:**

The copy number of mtDNA in various fat stores was higher in obese patients with type 2 diabetes than in obese patients without diabetes or in the control subjects and was related to the levels of leptin and proinflammatory cytokines.

**Electronic supplementary material:**

The online version of this article (10.1186/s12920-019-0486-7) contains supplementary material, which is available to authorized users.

## Background

Mitochondria are an attractive target for the prevention and treatment of obesity and its complications [1]. Mitochondria play a central role in the regulation of energy metabolism, signaling and apoptosis [2]. Recently, the biogenesis of mitochondria has been found to be enhanced by the action of nitric oxide (NO), which is a key signaling molecule in the regulation of vascular homeostasis, indicating that changes in the production or bioavailability of NO can be associated with the functioning of mitochondria [3]. However, the relationship between the signaling components of NO and mitochondria, as well as the metabolic disturbances of obesity in different tissues, is still not clear.

A disturbance in the regulation of energy metabolism in insulin-dependent tissues can be a key reason for the formation of insulin resistance [2, 3]. In particular, mitochondrial dysfunction has been shown to lead to oxidative stress, which initiates the development of atherosclerosis, cardiovascular diseases and insulin resistance [4, 5]. Changes in the level of mtDNA in peripheral blood leukocytes can also lead to oxidative stress and correlate with the development of metabolic syndrome [6]. Our previous data indicate a decrease in the number of copies of mtDNA in mononuclear blood cells (MNCs) and their increase in subcutaneous adipose tissue (SAT) [7].

In this regard, the aim of this study was to identify the relationship of the copy number of mtDNA in adipose tissues of different locations (SAT, mesentery (Mes), greater omentum (GO)), liver biopsy samples and MNCs with endothelial dysfunction markers (eNOS, endothelin-1 (ET-1), intercellular adhesion molecule 1 (iCAM-1), vascular cell adhesion molecule 1 (vCAM-1), vascular endothelial growth factor (VEGF)) and inflammatory mediators (TNF-α, IL-6, IL-8, C-reactive protein (CRP), leptin) in obese patients (body mass index (BMI) > 35 kg/m^2^) with and without type 2 diabetes.

## Materials and methods

### Study subjects

The study included 88 obese patients treated at the Kaliningrad Region Hospital. The control group consisted of 20 healthy donors (BMI = 22.3 ± 3.1 kg/m^2^) with anthropometric and biochemical measurements of lipid and carbohydrate metabolism within a healthy range.

Patients with abdominal obesity were ranked by the status of carbohydrate metabolism as follows: 54 patients with T2DM (BMI = 42.9 ± 8.45 kg/m^2^) and 34 patients without T2DM (BMI = 41.6 ± 4.5 kg/m^2^).

Control group and obese patients with and without type 2 diabetes were comparable sex- and age-wise.

The samples used for the study were peripheral venous blood (MNCs), adipose tissues (SAT, Mes and GO) and liver tissue. The patients were recruited into the study groups by the head of the Department of Reconstructive and Plastic Surgery, P.A. Zatolokin, MD, PhD, at the Regional Clinical Hospital of the Kaliningrad Region. Fat tissue biopsy samples were taken during routine laparoscopic operations in patients. The technical process of performing bariatric operations was standardized according to the methodical recommendations for bariatric surgeons (http://www.ifso.com/). RYGB was performed using the Lonroth technique. The materials used in the present study were venous blood samples drawn from fasting patients in the morning.

### Validation

The presence of obesity and T2DM was established on the basis of a detailed clinical and instrumental examination in a specialized hospital, guided by the World Health Organization (1999–2013) criteria for diagnosing diabetes and other types of hyperglycemia. Informed consent was signed by all patients. Verification of the diagnosis and recruitment of patients into the study groups were carried out at the Department of Reconstructive and Plastic Surgery on the basis of the regional clinical hospital in Kaliningrad.

Permission to conduct the study was obtained from the local ethics committee (Minute No. 4 of the meeting of the Local Ethics Committee at the Innovation Park of the Immanuel Kant Baltic Federal University, dated October 23, 2013).

### Blood chemistry

Mediators of carbohydrate and lipid metabolism (glucose, cholesterol, high-density lipoprotein (HDL), low-density lipoprotein (LDL), triglycerides, CRP) were measured on a CA-180 automatic biochemical analyzer (Furuno Electric Co., Ltd., Japan).

### Enzyme-linked immunosorbent assay

The concentrations of molecules (TNF-α, IL-6, IL-8, VEGF) were measured in plasma using the following sandwich enzyme-linked immunosorbent assays (ELISAs): Alfa-TNF-IFA-BEST, Interleukin-6-IFA-BEST, Interleukin-8-IFA-BEST, VEGF-IFA-BEST (Vector-Best kits, Russia; BIO Vendor, Czech Republic) in a Lazurite analyzer (Dynex Technologies Inc., USA). The concentrations of eNOS and endothelin-1 were measured in serum/plasma using an ELISA Kit for Nitric Oxide Synthase 3 Endothelial (NOS3) (Cloud-Clone Corp., USA) and Endothelin 1–21 kit (Biomedica, Austria). The concentrations of soluble circulating forms of iCAM-1 and vCAM-1 were measured in serum/plasma using a Human intercellular adhesion molecule 1, ICAM-1 ELISA Kit и and Human vascular cell adhesion molecule 1, VCAM-1 ELISA kit (Cusabio Biotech Co., Ltd., USA).

### Flow cytometry

Quantitative determination of leptin was evaluated by flow fluorimetry on a «Bio-Plex Protein Assay System» (Bio-Rad, USA) automated laser analyzer using Bio-Plex Pro Human Diabetes 10-Plex Assay commercial test system (Bio-Rad, USA). The method consists in the binding of the studied molecules with specific antibodies adsorbed on the surface of microspheres (magnetic granules), which allows to determine up to 100 analytes in one well. The results were read using an automatic Bio-Plex-200 System microplate photometer (Bio-Rad, USA) and the Bio-Plex Manager software (Bio-Rad, USA). Leptin concentrations were determined using a standard curve (determined dynamic range of 2–32,000 pg / ml) in accordance with the instructions of the manufacturer.

### Droplet digital PCR

DNA extraction from adipose tissues (SAT, Mes and GO), MNCs and liver tissue was carried out using a commercial QIAamp DNA Mini Kit (Qiagen, USA). The DNA concentration in the samples was measured with an Implen NanoPhotometer spectrophotometer. The mtDNA copy number was determined by ddPCR. The extracted DNA samples were pretreated with ApaI restriction endonuclease (New England Biolabs, Ipswich, USA). The components of ddPCR included a commercial 2x ddPCR Master Mix (Bio-Rad, Pleasanton, USA), fluorescent TaqMan probes, and oligonucleotide primers, and the reaction mixture was loaded into an automatic droplet generator (Bio-Rad, Pleasanton, USA).

The emulsified ddPCR mixture was transferred to standard 96-well plates and amplified in a thermocycler (Bio-Rad T100 thermal cycler) according to the following temperature protocol: 95 °C for 10 min, 40 cycles at 94 °C for 30 s and 53 °C for 60 s, and a final cycle at 98 °C for 10 min. After the amplification reaction, the strength of fluorescence was measured using a QX200 Droplet Reader (Bio-Rad, Pleasanton, CA). The data were analyzed with QuantaSoft program suite (version 1.7.4.0917). The absolute number of copies of mtDNA per cell was calculated using the methods outlined below, using formula:

$$ \mathrm{mtDNA}\ \mathrm{copy}\ \mathrm{number}\ \mathrm{per}\ \mathrm{cell}=\frac{2\times copies\ of\ mtDNA}{nuclear\  DNA\  copies} $$

### Statistical analysis

Verification of the normality of quantitative indicator distribution was carried out using the Shapiro-Wilk test. Because the investigated samples fit a normal distribution, the hypothesis of the equality of the mean sample values was verified using Student’s t-tests. To assess the significance of differences between independent quantitative samples that did not follow a normal distribution law, the nonparametric Kruskal-Wallis test was used. For detecting statistically significant differences between groups, a pairwise analysis was performed using the Mann-Whitney test. Differences were considered significant at the level of *p* < 0.05.

Correlations between the studied indices were determined using the Spearman correlation analysis and linear regression. For the analysis of an adequate linear regression model, the following regression residues were considered: the lack of autocorrelation of residues (Durbin-Watson test, *p*-values > 0.05), its normal distribution and the consistency of the dispersion residues (heteroscedasticity test, p-values < 0.05 were considered significant). R Statistical Software (version 3.3.1) was used to perform statistical analyses and generate graphs.

## Results

Patients with obesity have signs of lipid metabolism disorders in the presence and absence of type 2 diabetes. A higher level of LDL and a lower level of HDL were recorded in the group of patients with type 2 diabetes than in the control group. The levels of HDL and triglycerides were higher in patients without type 2 diabetes than in the controls (Table 1).

**Table 1:** Biochemical parameters of carbohydrate and lipid metabolism in blood serum in the studied patient groups

Biochemical indicators (mmol / l)	Control	Patients without type 2 diabetes	Patients with type 2 diabetes	*р*-value
Glucose	4.95 (1.5–5.25)	5.88 (5.6–6.62)	7.76 (5.84–9.84)*	< 0.001*
Cholesterol	4.60 (3.94–5.2)	5.33 (4.62–6.24)	5.34 (4.40–5.97)	0.027*
HDL	1.29 (0.97–2.46)	1.5 (1.18–4.75)*	1.09 (0.9–1.26)	< 0.001*
LDL	1.90 (1.54–2.46)	2.22 (1.15–2.80)	3.1 (2.44–3.52)*	< 0.001*
Triglycerides	1.3 (0.72–2.54)	2.09 (1.26–2.58)*	1.87 (1.30–2.14)	0.26

Note: the significance is determined using the Kruskel-Wallis test for several independent samples (* - *p* < 0.05); The Mann-Whitney criterion for two independent samples (* - *p* < 0.05); * - differences from the control

The content of CRP in serum in obese patients exceeded that of control subjects, reaching the maximal values in patients with type 2 diabetes (Table 1).

In all obese patients, an increase in the levels of proinflammatory molecules—IL-6, IL-8, and TNF-a - and a marker of metabolic abnormalities of leptin in blood plasma was observed with respect to those in the control subjects (Table 2). The concentrations of these factors were higher in patients with type 2 diabetes than in patients without diabetes (Table 2).

**Table 2:** The content of markers of endothelial dysfunction (eNOS, ET-1, iCAM-1, vCAM-1, VEGF) and inflammatory mediators (TNF-α, IL-6, IL-8, CRP, leptin) in the serum of patients

Mediators	Control	Patients without type 2 diabetes	Patients with type 2 diabetes	*р*-value
Nitrite (pg / ml)	3.57 (3.08–4.38)	3.68 (3.1–4.04)	4.03 (3.28–5.13)	0.5471
Endothelin-1 (pmol / L)	0.24 (0.17–0.32)	1.04 (0.32–4.16)*	0.49 (0.20–1.04)	0.02086*
iCAM-1 (pg / ml)	5667 (5117–5930)	5675 (5428–6058)	5856 (5450–6159)	0.4578
vCAM-1 (ng / ml)	11.03 (9.69–11.78)	11.63 (9.01–30.46)	33.76 (11.32–82.31)*	0.01768*
VEGF (pg / ml)	359.2 (249.3–468.9)	389.5 (181.7–570.3)	439.3 (264.2–625.4)	0.5606
TNF-a(pg / ml)	3.3 (2.65–4.77)	13.44 (8.21–14.94)*	32.6 (25.9–35)*	< 0.001*
IL-6 (pg / ml)	1.35 (0.52–1.75)	3.06 (2.36–3.71)*	4.46 (3.53–6.16)*	< 0.001*
IL-8 (pg / ml)	6.5 (4.7–9.7)	9.0 (7.54–10.62)*	9.19 (7.64–11.10)*	0.001429*
CRP(mmol / l)	3.56 (1.77–8.65)	13.39 (9.95–15.99)*	8.06 (3.54–14.23)*	0.002*
Leptin (ng / ml)	4.27 (2.90–8.20)	28.73 (18.1–49.9)*	40.83 (11.41–55.05)*	< 0,001*

Note: the significance is determined using the Kruskel-Wallis test for several independent samples (* - *p* < 0.05); The Mann-Whitney criterion for two independent samples (* - *p* < 0.05); * - differences from the control

An increase in the copy number of mtDNA in the adipose tissues of the Mes and SAT and a decrease in mtDNA in MNCs were observed in the group of patients with type 2 diabetes compared with those in the control group. Furthermore, an increase in the copy number of mtDNA in the GO and a decrease in mtDNA in liver cells were found in patients with type 2 diabetes compared to those in patients without type 2 diabetes. However, the copy number of mtDNA in all studied tissues in patients without type 2 diabetes did not differ from that of the control subjects.

## Discussion

With obesity, the systemic nature of chronic subclinical inflammation is established [8].

**Fig. 1:**
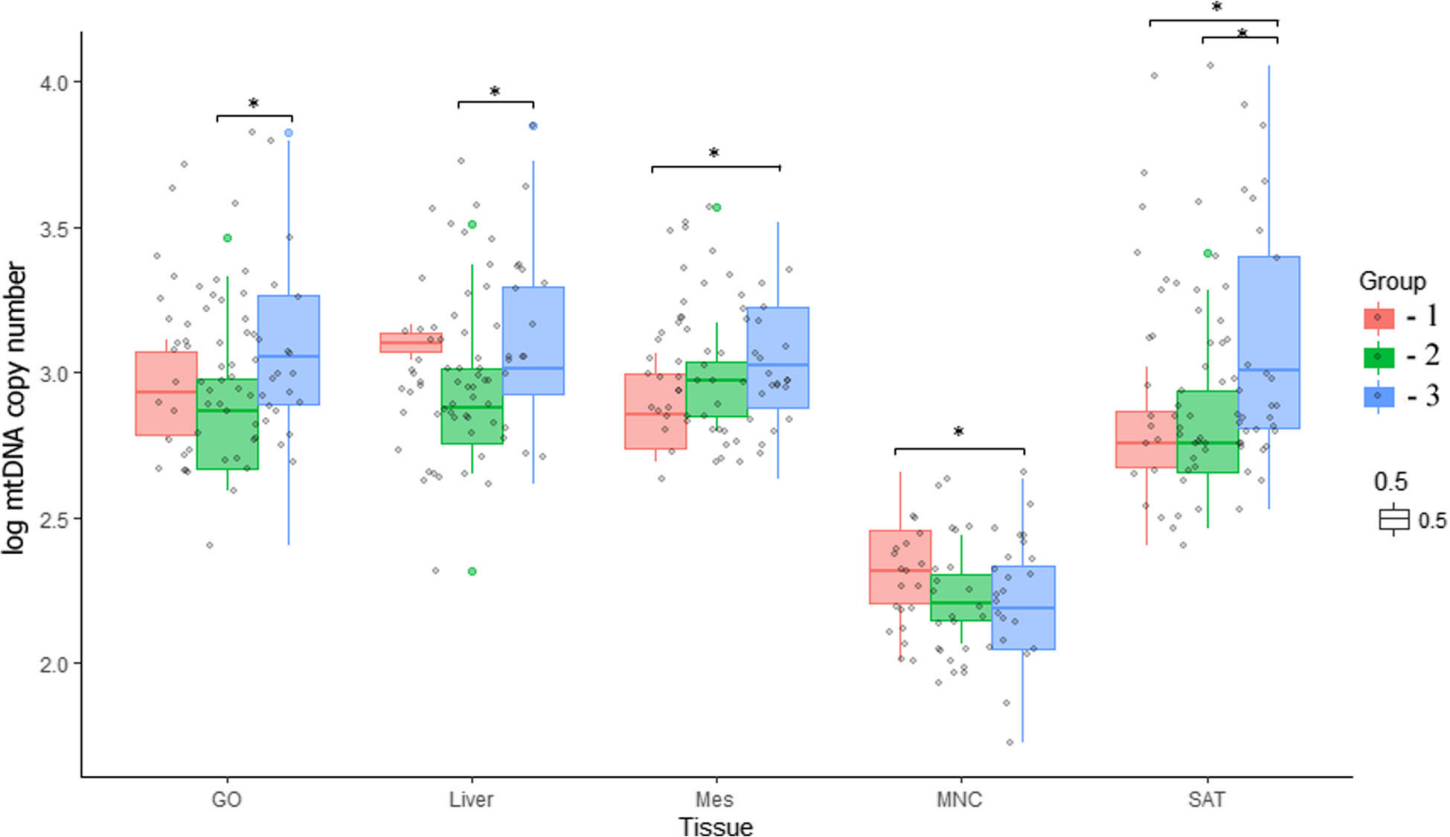
The number of mtDNA in adipose tissue of different locations, liver biopsy and MNC in the study patient groups: 1 - control group, 2 - obese patients without type 2 diabetes, 3 - obese patients with type 2 diabetes. Note: Copies per cell, the significance is determined using the Mann-Whitney criterion for two independent samples (* - *p* < 0.05)

The plasma levels of the proinflammatory cytokines TNF-a, IL-6 and IL-8 were increased in all obese patients and were positively interrelated (Fig. 1) (Table 2). Moreover, the levels of these factors were positively correlated with BMI, serum glucose and cholesterol levels, and levels of TNF-a and IL-6 were correlated with serum LDL concentrations. The correlations we found can indicate the predominant role of proinflammatory mediators in the formation of disturbances in carbohydrate and lipid metabolism in obese patients with and without type 2 diabetes (Fig. 1, Table 2).

In the literature, the data are ambiguous with regard to changes in the number of copies of mtDNA in adipose tissues in obese patients [9]. In our study, the content of mtDNA in different fat stores was higher in obese patients with type 2 diabetes than in obese patients without diabetes or in control subjects.

We assume that the increase in the number of copies of mtDNA in the studied biopsy specimens of adipose tissues can be considered a compensatory mechanism aimed at maintaining normal biogenesis of mitochondria under conditions of oxidative stress mediated by a high concentration of proinflammatory cytokines. With the damaging effect of reactive oxygen species (ROS) during oxidative stress, the mechanism of replication of the mitochondrial genome and division of the organelle is triggered [10]. The above is supported by positive correlations between the number of mtDNA in SAT and the levels of proinflammatory cytokines IL-6 (r = 0.3) and TNF-a (r = 0.4) and negative correlations between mtDNA in MNCs and levels of IL-6 (r = − 0.42) and TNF-a (r = − 0.33) (*p* < 0.05) (Additional file 1).

Mitochondria are involved in the regulation of carbohydrate metabolism and contribute to the pathogenesis of type 2 diabetes in obesity [11]. The number of mtDNA in GO and SAT was positively correlated with the indices of carbohydrate and lipid metabolism—glucose level (r = 0.39, r = 0.34, respectively) and LDL cholesterol (*r* = 0.37, *r* = 0.32, respectively) (*p* < 0.05). In addition, mtDNA in GO was negatively correlated with HDL cholesterol (*r* = − 0.27, *p* < 0.05). In MNCs, excellent patterns were noted: the number of mtDNA correlated negatively with glucose level (*r* = − 0.27) and leptin (*r* = − 0.52) (*p* < 0.05). High plasma contents of the leptin hormone are known to be a frequent companion of obesity and a sign of metabolic disorders in individuals with obesity [12], which was confirmed in our study; the plasma leptin level was increased in obese patients with and without type 2 diabetes and positively correlated with the levels of proinflammatory cytokines (TNF-a, IL-6 and IL-8), glucose and vCAM-1, that indicate a negative role of the increase in the number of mtDNA in carbohydrate and lipid metabolism.

The high content of free fatty acid (FFA), which is formed in excess during lipolysis in obesity, contributes to the development of oxidative stress in adipose tissues cells [13]. The effect of FFA on endothelial cells worsens insulin-stimulated activation of eNOS [14], reducing the bioavailability of NO and enhancing oxidative stress. In our study, the level of LDL was positively correlated with the markers of endothelial dysfunction, nitrites (*r* = 0.32) and endothelin-1 (*r* = 0.35) (*p* < 0.05).

Another sign of impaired endothelial function is elevated levels of circulating soluble iCAM-1 and vCAM-1 in blood [15–17], which is partially reflected in our study: the vCAM-1 level was elevated in obese patients with type 2 diabetes. The content of the circulating adhesion molecule vCAM-1 was positively correlated with the levels of CRP (*r* = 0.42), leptin (*r* = 0.31), TNF-a (*r* = 0.35) and IL-6 (*r* = 0.52), indicating the participation of proinflammatory molecules in the formation of endothelial dysfunction (*p* < 0.05). In patients with obesity without type 2 diabetes, an increase in the concentration of endothelium-1 was found compared with that in the control. Positive correlations were found between the levels of endothelin-1 (*r* = 0.39), iCAM-1 (*r* = 0.45) and vCAM-1 (*r* = 0.41) and glucose content (*p* < 0.05). In addition, in the group of obese patients with type 2 diabetes, a correlation was found between the level of endothelium-1 and the number of mtDNA in SAT (*r* = 0.61, *p* < 0.05). Thus, we registered signs of the development of endothelial dysfunction in all obese individuals surveyed.

The limitation of this study is the small spectrum of the studied molecular markers of metabolic disorders and the small number of examined patients. The change in the copy number of mtDNA in various tissues could be suggested as an early marker of metabolic abnormalities.

## Conclusions

1. In patients with obesity (BMI > 35 kg/m^2^), regardless of the presence/absence of type 2 diabetes, systemic subclinical inflammation and signs of endothelial dysfunction were revealed.
2. The increase in the copy number of mtDNA in the test tissues was interrelated with the increase in the content of proinflammatory cytokines IL-6, IL-8 and TNF-a in blood plasma in all the groups of subjects studied.
3. The increase in the copy number of mtDNA in the studied tissues was interrelated with the disturbance in carbohydrate and lipid metabolism, as well as the increase in the content of endothelium-1 in obese patients with type 2 diabetes.
4. The increase in the copy number of mtDNA in adipose tissues (GO, SAT) and MNC was interrelated with a disturbance in carbohydrate and lipid metabolism.

## Additional file

Additional file 1: **Table S3**. Correlation interrelations of the studied metabolites. (DOCX 100 kb)

## Abbreviations

BMI: Body mass index
CRP: C-reactive protein
ddPCR: Droplet Digital PCR
eNOS (NOS3): Endothelial nitric oxide synthase
ET-1: Endothelin-1
FFA: Free fatty acid
GO: Greater omentum
HDL: High-density lipoproteins
iCAM-1: Inter-Cellular Adhesion Molecule 1
IR: Insulin resistance
LDL: Low-density lipoprotein
Mes: Mesentery of the small intestine
MNC: Blood mononuclear cells
mtDNA: Mitochondrial DNA
NO: Nitric oxide
ROS: Reactive Oxygen Species
SAT: subcutaneous adipose tissue
vCAM-1: Vascular cell adhesion molecule 1
VEGF: Vascular endothelial growth factor
