# Supplementary figures and images for: The relationship between the mtDNA copy number in insulin-dependent tissues and markers of endothelial dysfunction and inflammation in obese patients
Source: BMC Med Genomics. 2019 Mar 13;12(Suppl 2):41. doi: 10.1186/s12920-019-0486-7 (PMC6416834; doi:10.1186/s12920-019-0486-7)

Additional file 1

Table S3. Correlation interrelations of the studied metabolites


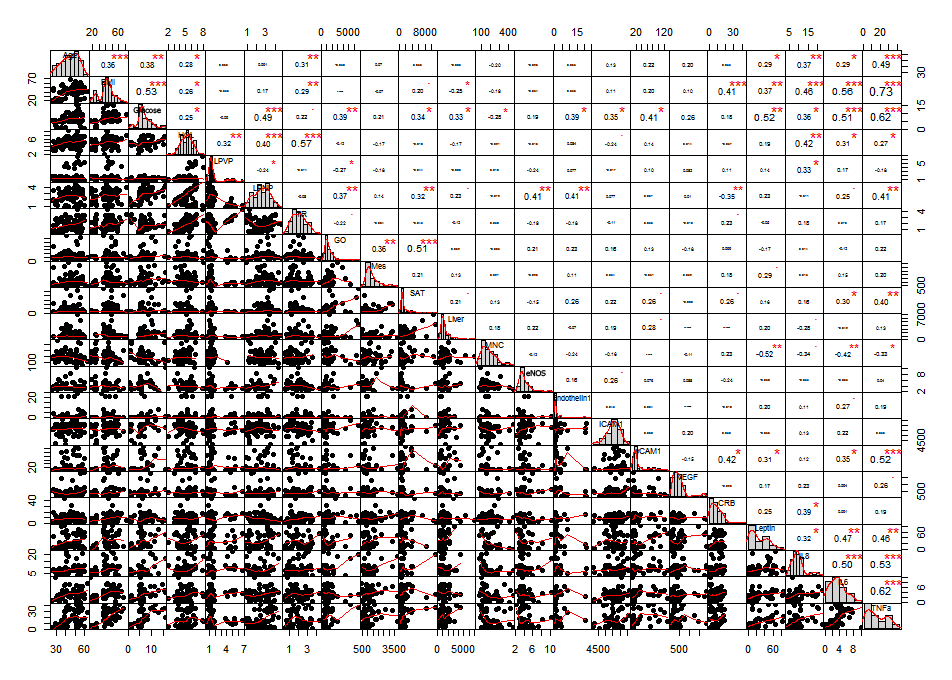

Supplement: Supplementary file 1 — Table S3. Correlation interrelations of the studied metabolites. (DOCX 100 kb) [file 12920_2019_486_MOESM1_ESM.docx]
